# Epidemiology and Outcomes of Bloodstream Infections in Patients With Solid Tumors in a Central American Population at Mexico Hospital, San Jose, Costa Rica

**DOI:** 10.1200/JGO.17.00058

**Published:** 2017-12-15

**Authors:** Jorge Calvo-Lon, Denis U. Landaverde, Allan Ramos-Esquivel, Juan M. Villalobos-Vindas

**Affiliations:** **Jorge Calvo-Lon** and **Juan M. Villalobos-Vindas**, Mexico Hospital; **Denis U. Landaverde**, Mexico Hospital and University of Costa Rica; and **Allan Ramos-Esquivel**, San Juan de Dios Hospital and University of Costa Rica, San Jose, Costa Rica.

## Abstract

**Purpose:**

Bloodstream infections (BSIs) are an important cause of mortality in patients with solid tumors. We conducted a retrospective study to evaluate the epidemiologic profile and mortality of patients with solid tumors who have BSIs and were admitted to Mexico Hospital. This is the first study in Costa Rica and Central America describing the current epidemiologic situation.

**Methods:**

We analyzed the infectious disease database for BSIs in patients with solid tumors admitted to Mexico Hospital from January 2012 to December 2014. Epidemiology and mortality were obtained according to microorganism, antibiotic sensitivity, tumor type, and presence of central venous catheter (CVC). Descriptive statistics were used.

**Results:**

A total of 164 BSIs were recorded, the median age was 58 years, 103 patients (63%) were males, and 128 cases of infection (78%) were the result of gram-negative bacilli (GNB). *Klebsiella pneumoniae* (21%), *Escherichia coli* (21%), and *Pseudomonas aeruginosa* (15%) were the most common microorganisms isolated. Gram-positive cocci (GPC) were found in 36 patients, with the most frequent microorganisms being *Staphylococcus aureus* (10%) and *Staphyloccocus epidermidis* (6%). With respect to tumor type, BSIs were more frequent in the GI tract (57%) followed by head and neck (9%) and genitourinary tract (8%). Regarding antibiotic susceptibility, only 17% (GNB) expressed extended-spectrum beta-lactamase and 12% (GPC) had methicillin resistance. Patients with CVCs (n = 59) were colonized mainly by GNB (78%). Overall the mortality rate at 30 days was about 30%.

**Conclusion:**

GNB are the most frequent cause of BSIs in solid tumors and in patients with CVCs. GI cancers had more BSIs than other sites. Mortality and antibiotic sensitivity remained stable and acceptable during this observational period in this Latin American population.

## INTRODUCTION

According to the World Health Organization, cancer is a leading cause of death worldwide, with approximately 14 million new cases annually.^1^ Patients with cancer often experience several types of complications during their treatments, including bloodstream infections (BSIs), which are a common cause of morbidity and mortality.^2^ Most of the published data on BSIs in patients with cancer are from patients with hematologic malignancies^3^; information is scarce on BSIs in solid tumors worldwide and even less is available for patients in Latin America.^4^ We conducted a retrospective study to evaluate the epidemiologic profile and mortality of patients with solid tumors who had BSIs and had been admitted to Mexico Hospital. To the best of our knowledge, this is the first study that describes the current epidemiologic situation in Costa Rica and Central America.

## METHODS

This is a retrospective study conducted at Mexico Hospital in San Jose, Costa Rica. We included all hospitalized patients with solid tumors who had BSIs who were entered into our infectious disease database from January 1, 2012, to December 31, 2014. Patients with hematologic malignancies were excluded. We analyzed the episodes of BSIs according to demographic characteristics, microorganism, antimicrobial sensitivity, tumor type, and the presence of a central venous catheter (CVC). This study was approved by the local ethics committee.

Centers for Disease Control and Prevention criteria for defining infections were used,^5,6^ as well as the Third International Consensus definitions for sepsis and septic shock (Sepsis-3).^7^

BSI was defined as the presence of a microorganism in the blood, isolated by at least one positive blood culture that must have occurred close to or concomitant with a clinically or laboratory-proven site of infection. For a definitive diagnosis of intravascular catheter-related BSIs, the same microorganism was required to be isolated from at least one percutaneous blood culture of the intravascular catheter according to the updated Infectious Diseases Society of America clinical practice guidelines for the diagnosis and management of intravascular catheter-related infections.^8^

Blood samples were inoculated into BacT/ALERT FA (bioMérieux, Durham, NC) culture bottles and incubated in a BacT/ALERT 3D automated continuous monitoring system until microbial growth became evident or for 6 days. Bacteria were identified and antibiotic susceptibility was obtained by using the automated Vitek 2 System (bioMérieux). Clinical and Laboratory Standards Institute breakpoints and rules were used to determine antimicrobial susceptibility.^9^ The antibiotics used for antimicrobial susceptibility testing for gram-negative bacilli (GNB) were amikacin, nalidixic acid, ampicillin/sulbactam, cefalotin, cefotaxime, ceftazidime, ciprofloxacin, gentamicin, piperacillin/tazobactam, nitrofurantoin, and trimethroprim/sulfamethoxazole. Those used for gram-positive bacilli were ampicillin, ciprofloxacin, levofloxacin, linezolid, minocycline, nitrofurantoin, teicoplanin, tetracycline, trimethroprim/sulfamethoxazole, and vancomycin.

### Statistical Analysis

Because of the retrospective nature of this study, all patients with solid tumors who had BSIs were included; during the observational time period, neither prespecified sample sizes nor pre-established hypotheses were available for evaluation. Categorical variables are presented as percentages and were compared by the χ^2^ test when appropriate. Continuous variables are presented as the mean ± standard deviation. Mortality rate was defined as the number of all causes of death within 30 days after the date of documented BSI divided by the total number of BSIs in the same period. Data were analyzed by using SPSS for Mac version 20.0 (SPSS, Chicago, IL).

## RESULTS

We used our biostatistical database to review 1,210 patients who were admitted to the Mexico Hospital and were diagnosed with malignant solid tumors from January 1 to December 31, 2012. One hundred sixty-four episodes of BSIs were adequately documented, and 14% of hospitalized patients with solid tumors experienced at least one episode of BSI. The annual distribution is summarized in Table 1. The median age was 57.9 years (range, 15 to 88 years). One hundred four cases (63%) of BSIs occurred in males, and 33 patients (20%) had febrile neutropenia.

**Table 1:**
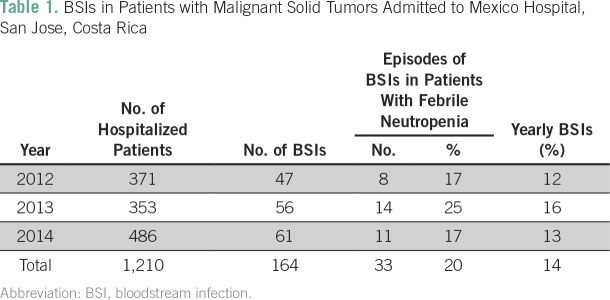
BSIs in Patients with Malignant Solid Tumors Admitted to Mexico Hospital, San Jose, Costa Rica

Table 2 shows the distribution by year of BSIs according to microorganism. A statistically significant predominance of GNB over gram-positive cocci (GPC) was found (78% *v* 22%; χ^2^ *P* = .029). Overall, the most frequent GNB were *Escherichia coli* (21.3%), *Klebsiella pneumoniae* (21.3%), and *Pseudomonas aeruginosa* (15.2%). The most frequent gram-positive organisms were *Staphylococcus aureus* (9.8%), *Staphyloccocus epidermidis* (5.5%), and all *Streptococci* species together (3%). Among neutropenic patients, GNB were more common than GPC (81% *v* 19%, respectively; Table 3). Table 4 describes antibiotic susceptibility.

**Table 2:**
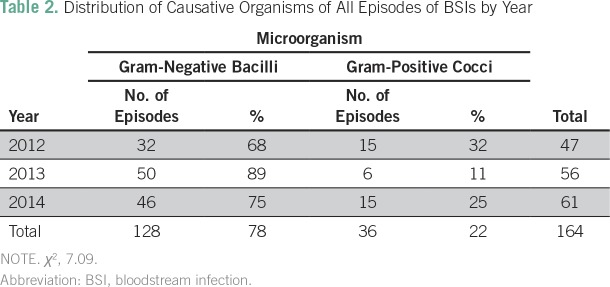
Distribution of Causative Organisms of All Episodes of BSIs by Year

**Table 3:**
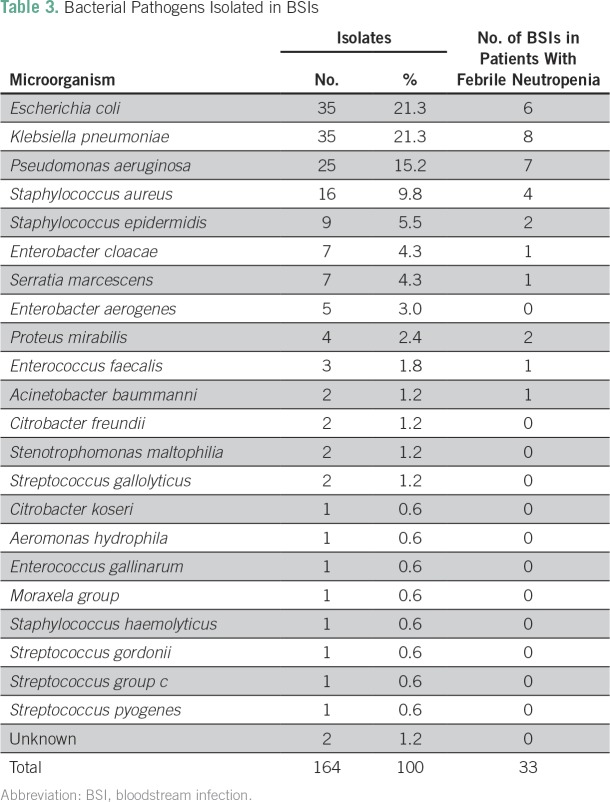
Bacterial Pathogens Isolated in BSIs

**Table 4:**
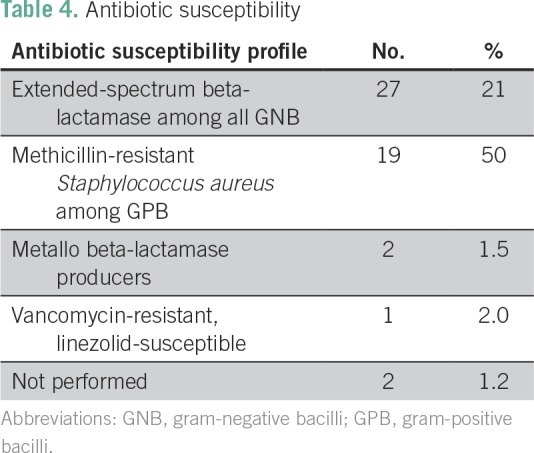
Antibiotic susceptibility

Most of the patients (n = 105 [64%]) had peripheral venous catheters, 45 (27%) had short-term CVCs, and 14 (9%) had implantable ports. There was no statistical difference in terms of the BSIs detected and the type of venous catheter inserted or the site of insertion (χ^2^ *P* = .27); however, in patients with CVCs, GNB are the prevalent microorganisms.

We found that BSIs occurred more frequently in patients with GI tumors; 93 patients (57%) had gastric cancer, colorectal cancer, or no colorectal carcinomas, and 15 (9%) had head and neck carcinomas (Table 5). Mortality rate at 30 days remained stable during the observational period, being on average 30%.

**Table 5:**
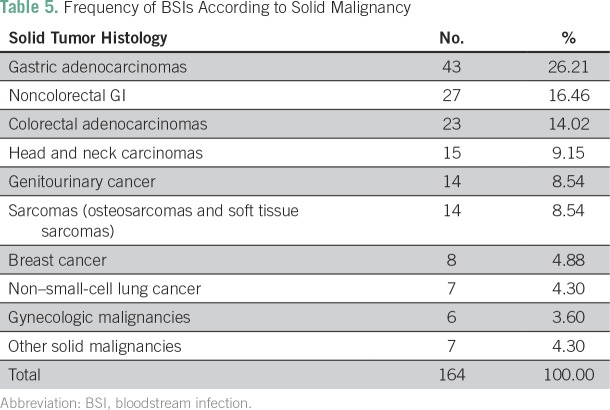
Frequency of BSIs According to Solid Malignancy

## DISCUSSION

Little information has been reported about BSIs in hospitalized patients with solid tumors worldwide. As far as we know, there are no published data for either Costa Rica or Central America, and even less is known regarding the epidemiology and mortality of BSIs in our region. Most of the data on BSIs have been extrapolated from studies on hematologic malignancies in Europe and the United States.^10-12^

One interesting finding is that 14% of hospitalized patients with solid tumors in Mexico Hospital developed a BSI, ranging from 1.2 to 1.6 cases per 1,000 admissions of patients with solid malignancies per year. In the United States, the incidence of sepsis in patients with cancer in this setting is about 16.4 (including hematology patients); in the United Kingdom, 3.6 per 1,000 admissions per year; and in Spain, 0.95 per 1,000 admissions per year^13-15^; thus, the number of epidemiologic events in our population is similar to that in Spain.

GNB were by far the most frequent causative agents isolated in BSIs in our patients (78% of the episodes). This was statistically significant when compared with GPC (*P* = .029). In the last two decades, GPC were the leading cause of BSIs in patients with cancer (including those with hematologic malignancies), but several institutions have recently reported that up to 65% of BSIs are the result of GNB.^3^ We have observed the same epidemiologic trend, with *K. pneumoniae*, *E. coli*, and *P. aeruginosa* being the main microorganisms involved in BSIs, and the same microorganisms that are responsible for bacteremia in other series reported in Europe.^4,12,16^ This high incidence of GNB can be attributed to the fact that most of the bacteremias occur after surgery in the GI tract.^17^ In our series, we found that about 20% of the BSIs occurred in neutropenic patients, with gram-negative rods being the most commonly isolated, in concordance with other reports mainly from developing countries.^18^ In terms of susceptibility profile, 68% of the bacteria were susceptible to the majority of antibiotics tested, and 21% expressed extended-spectrum beta-lactamase; *E. coli* and *K. pneumoniae* isolates that produced extended-spectrum beta-lactamase ranged from 12% to 75% (mean, 35%) in patients with cancer (including those with and without febrile neutropenia).^19^

Of the 59 patients with bacteremia and CVCs, 43 (73%) experienced BSIs associated with GNB, which is concerning because gram-positive microorganisms are more frequently described as the causative agents in catheter-related bacteremia.^20^ This increased prevalence of gram-negative bacteria could be attributed to inadequate handling of CVCs by health care personnel. There was no statistical difference between type of catheter and the site of insertion, which means that regardless of the type of CVC (long-term or short-term), the incidence of catheter-related BSIs was the same and was mainly due to GNB. This information is particularly useful for prescribing empirical antibiotics. In our case, we should start antibiotics with an anti-GNB spectrum as first-line therapy, either including or omitting anti-GPC treatment.

With respect to tumor site, 57% of BSI episodes occurred in GI cancers, followed by head and neck cancers (9.2%) and genitourinary cancers (8.5%). This distribution may explain the high incidence of BSIs caused by GNB, because this population underwent surgery, and bacteria may have translocated from the GI tract. In other studies, gram-negative BSIs were reported in about 62% of the patients in this setting.^17^

Our mortality rate at 30 days was about 30%, and the mortality rate ranged from 16% to 30% in the United States, Brazil, and Spain.^3,11^ On the basis of those data, we had an acceptable and stable mortality rate during the observation time period.

Our study has some limitations. First, it was based on a retrospective database, and we cannot exclude any selection bias. In addition, we cannot rule out any misclassification bias resulting from the retrospective collection of the data. Furthermore, our findings are based on a single institution experience, and the external validity of our research could be limited. Despite these caveats, we think our findings increase the knowledge about this important topic in our area.

In conclusion, limited data are available in Latin America regarding BSIs in patients with solid malignancies. With this study, we were able to determine that GNB are by far the most frequent cause of BSIs in our population. Neutropenic patients with solid tumors are infected mainly by GNB. BSIs are more frequent in patients with GI tumors. In our environment, infected CVCs are due to GNB. Mortality and antibiotic susceptibility remain stable and acceptable in this Latin American population. Understanding the epidemiology in our area can improve and optimize first-line antimicrobial therapies.
